# One-step real-time multiplex reverse transcription-polymerase chain reaction assay with melt curve analysis for detection of potato leafroll virus, potato virus S, potato virus X, and potato virus Y

**DOI:** 10.1186/s12985-021-01591-3

**Published:** 2021-06-29

**Authors:** Nobuya Onozuka, Takehiro Ohki, Norikuni Oka, Tetsuo Maoka

**Affiliations:** 1 Division of Agro-Environmental Research, Hokkaido Agricultural Research Center, National Agriculture and Food Research Organization (NARO), Hitsujigaoka 1, Toyohira, Sapporo, Hokkaido 062-8555 Japan; 2 Graduate School of Agriculture, Hokkaido University, Kita 8, Nishi 5, Kita-ku, Sapporo, Hokkaido 060-0808 Japan; 3 Department of Regional Strategy, Hokkaido Agricultural Research Center, National Agriculture and Food Research Organization (NARO), Hitsujigaoka 1, Toyohira, Sapporo, Hokkaido 062-8555 Japan

**Keywords:** Potato viruses, Melt curve analysis, High-throughput detection, Bulked test

## Abstract

**Background:**

Certification of seed potato as free of viruses is essential for stable potato production. Among more than 30 virus species infecting potato, potato leafroll virus (PLRV), potato virus S (PVS), potato virus X (PVX), and potato virus Y (PVY) predominate worldwide and should be the targets of a high-throughput detection protocol for seed potato quarantine.

**Results:**

We developed an assay based on one-step real-time multiplex reverse transcription-polymerase chain reaction (mRT-PCR) with melt curve analysis for the four viruses and one internal control, potato elongation factor 1 alpha gene (*EF1α*). Virus-specific primers were derived from conserved regions among randomly selected representatives considering viral genomic diversity. Our assay simultaneously detected representative Japanese isolates of PLRV, O lineage of PVS, PVX, and NTN strain of PVY. The variability of melting temperature (Tm) values for each virus was confirmed using Japanese isolates, and virus species could be identified by the values of 87.6 for PLRV, 85.9 for PVX, 82.2 (Ordinary lineage) to 83.1 (Andean lineage) for PVS, and 79.4 (NA-N strain) to 80.5 (O strain and NTN strain) for PVY on average. The reliability of calculation was validated by comparing the calculated Tm values and measured Tm values and the values had a strong linear correlation (correlation of determination: *R*^2^ = 0.9875). Based on the calculated Tm values, representative non-Japanese isolates could also be identified by our assay. For removing false positives, two criteria were set for the evaluation of result; successful amplification was considered as 30.0 ≥ threshold cycle value, and the virus-specific peak higher than the *EF1α*-specific peak was considered as positive. According to these criteria, our assay could detect PLRV and PVS from 100-fold dilution of potato leaf homogenate and PVX and PVY from 1000-fold in a model assay.

**Conclusion:**

This new high-throughput detection protocol using one-step real-time mRT-PCR was sensitive enough to detect viruses in a 100-fold dilution of singly-virus contaminated homogenate in a model assay. This protocol can detect the four viruses in one assay and yield faster results for a vast number of samples, and greatly save the labor for seed potato quarantine and field surveys.

**Supplementary Information:**

The online version contains supplementary material available at 10.1186/s12985-021-01591-3.

## Background

Potato (*Solanum tuberosum* L.), the fourth most important crop in the world [1], is propagated using tubers and susceptible to more than 30 virus species [2]. In Japan, four virus species of them are mainly found in potato fields: potato leafroll virus (PLRV), potato virus S (PVS), potato virus X (PVX), and potato virus Y (PVY). These four viruses also are economically important worldwide because these viruses are distributed in most potato-planting areas and reduce the quality and quantity of the yield [3].

For controlling virus diseases in potatoes, advanced nations have seed potato certification programs to test seed potato for viruses and grade seed potato lots by their virus-contamination level [4–8]. Enzyme-linked immunosorbent assay (ELISA) has been used in such programs; however, many pathogens, including viruses, bacteria, nematodes, and fungi, infect potatoes, such that high-throughput assays alternative to ELISA are required for certification programs. Toward meeting this need, multiplex reverse transcription-polymerase chain reaction (mRT-PCR)-based assays are becoming common [9–12]. We have already developed one-step conventional mRT-PCR assays, combined with a filter-paper-based simple RNA preparation for the four major potato viruses and a plant gene as internal control [13]. The advantage of this protocol is that as cost-effective as the ELISA it yields results more rapidly; however, the subject is still time-consuming for the electrophoretic analysis and the risk of PCR products contamination by open-tube manipulation.

As a high-throughput alternative, one-step real-time mRT-PCR is a desirable candidate. The real-time PCR technique is classified into two methods, one method using fluorescent-labeling probes and the other using double-stranded DNA (dsDNA) binding fluorescent dye. The latter, the intercalator method, is more cost-effective because only two fluorescent dyes, a dsDNA binding fluorescent dye and a reference dye for each well’s standardized fluorescent intensity, need to be compared with fluorescent-labeling probes to each target in the former method. In the intercalator method, the DNA dye is captured to dsDNA, not to single-stranded DNA (ssDNA), in a target non-specific manner, and amplicons can be identified by the melting temperature (Tm), which is measured as the release of the dye during denaturing dsDNA to ssDNA (melt curve analysis). Among intercalator methods, only one assay, which can identify multiple amplicons of potato viruses with different Tm values by the melt curve analysis, was reported [12]. Their assay could simultaneously detect five viruses: PLRV, potato virus A (PVA), PVS, PVX, and PVY using Evagreen DNA dye. Although their strategy can be used to detect the four major potato viruses, the reactivity of the assay for PVS and PVY must be reconsidered. Lineages of PVS and strains of PVY were reclassified based on genomic sequences since they had reported the assay. PVS was divided into three lineages: Ordinary lineage (PVS^O^), Andean lineage (PVS^A^), and Phureja lineage (PVS^P^) [14]. PVY is more complex and divided into five nonrecombinant strains and numerous recombinant strains [15]. In addition, reverse transcription and real-time PCR were performed separately in their protocol [12], so their assay would be improved by applying a one-step real-time RT-PCR assay.

Here, we developed a new one-step real-time mRT-PCR assay for the four major potato viruses and used a plant gene as the internal control. Combined with the simple RNA preparation method [13], the detection of Japanese isolates was demonstrated. The reliability of calculating Tm values were confirmed by comparing measured and calculated Tm values for Japanese representatives. The variability of Tm values for each virus species was validated by the calculated Tm values for representatives, which were randomly selected considering viral genomic diversity. The detection sensitivity for each virus was confirmed using potato leaves that were singly-infected a Japanese isolate of each virus.

## Materials and methods

### Virus resources

All viral isolates in the previous report were also used in this study [13]. The viral isolates were propagated in mechanically inoculated potato plant cv. Irish Cobbler (IC) and virus infection was confirmed by conventional RT-PCR [13]. Virus-infected leaves were stored at − 80 °C until use.

### Primer design

Primers for PLRV, PVS, PVX, PVY were designed based on conserved regions among more than six representative sequences (Additional file 1: Figure S1). The viral genome sequences were randomly retrieved from the NCBI sequence database (https://www.ncbi.nlm.nih.gov/), considering lineages of PVS and strains of PVY. Alignment analysis was performed using the MEGA-X program (https://www.megasoftwa re.net/) to determine the conserved regions. For internal control, which was an indicator of successful RNA preparation and DNA amplification, five sequences of mRNA for potato elongation factor 1 alpha gene (*EF1α*) (GenBank accessions: DQ2288628, DQ222490, AJ536671, AB061263, and KF537426) were used and the conserved region was also determined using MEGA-X. The melting temperature (Tm) value of primer was calculated by Oligo calculator (http://www.ngrl.co.jp/tools/0217oligocalc.htm), and all primers were designed to be around 60 °C in their Tm values, except degenerated primers for PVS which were resulted in the range of 60–65 °C. The Tm values for predicted amplicons were calculated by uMelt batch software of DNA-UTAH.ORG (https://dna-utah.org/index.html) with the following options: 30 mM mono cation, 1.2 mM free Mg^2+^, 10% dimethyl sulfoxide (DMSO), “Huguet et al. [16]” thermodynamic library and “Medium (0.5 °C)” resolution. In the preliminary examination, primers were designed without adjusting the options, resulting in that measured Tm values were far from calculated values and overlapped with each other. The options were adjusted to make the difference between calculated and measured Tm values less than 1.0 °C using the result to predict precisely amplicon Tm values for primers.

### Optimization of one-step real-time mRT-PCR assay

For this experiment, isolates ChLR_2 for PLRV, M for PVS^O^, O-IC249 for PVX, and Eu-12Jp for NTN strain of PVY (PVY^NTN^) were used. Primer concentrations were optimized by comparing the heights of each specific peak in the melt curve for the four viruses contaminated samples to adjust the amplification efficiency for simultaneous detection of all four viruses. All primer concentrations were initially set at 0.2 µM in the reaction mixture and decreased or increased depending on the height of peaks. For simultaneous detection of the four viruses, the RNA solution was prepared from a potato leaf homogenate containing all four viruses, prepared by mixing an equal volume of four potato leaf homogenates, each containing one of the four viruses.

PCR Procedure was as follows. The RNA solution was extracted using a simple filter paper-based method [13]. The RNA solution was stored at − 80 °C until use, and 1 µl of the supernatant was used as the template. Using PrimeScript One-Step RT-PCR Kit Ver.2 (Takata Bio, Shiga, Japan), one-step real-time mRT-PCR was performed in 10 μl of the reaction mixture, which contained 1 µl of the RNA solution, 1 × EvaGreen (Biotium Inc., CA, USA), 0.1 × ROX Reference Dye (Thermo Fisher Scientific, Waltham, MA, USA), 1 × 1 Step buffer and 1 × PrimeScript 1 Step Enzyme Mix. A real-time PCR system, QuantStudio 3 (Thermo Fisher Scientific), was programmed for each step shown in Table 1, using the instrument software, Design and Analysis New (DA2). The results, including amplification plot and melting curve, were obtained and analyzed with the software.

**Table 1:** Optimized RT-PCR program using Quant Studio 3

Process	RT^c^		PCR (40cycle)		Melting curve analysis			
Subprocess	cDNA synthesis	Denature	Denature	Annealing-Elongation	Denature	Annealing	Melting^d^	Hold
Tmp^a^	50 °C	95 °C	95 °C	58 °C	95 °C	60 °C	0.03 °C/s	95 °C
Time	5 min	10 s	5 s	31 s	15 s	15 s		1 s
Measure^b^				●			●	

^a^Temperature

^b^Measure fluorescent intensity in the steps marked with black dot (●)

^c^Reverse transcription

^d^Temperature was increased 0.03 °C per second in this step to dissociate dsDNA gradually

A non-specific peak was observed in the melt curve for virus-free potato leaf and similar to the PVS-specific peak in the multiple detections. The assay was performed to detect three viruses without PVS genome or PVS primers to reveal whether the peak in multiple detections was a specific or non-specific product. For the assay without PVS genome, RNA solutions were prepared from each singly-virus-infected leaf homogenate, except PVS-infected, and an equal volume of the three solutions was mixed and used as a template. For the assay without PVS primers, the template solution was prepared from the mixture of the four potato leaf homogenates. To validate the calculation, calculated Tm values for Japanese isolates in Table 2 were compared with measured Tm values, and the correlation of them was confirmed by the correlation of determination (*R*^2^) for the regression line, which was given by the spreadsheet program, Microsoft Excel 365. The calculation of Tm values for non-Japanese isolates was performed as described above.

**Table 2:** Primers information and amplicons Tm values

Target	Polarity	Primer Sequence (5'–3')^a^	Final conc. (µM)	Amplicon size (bp)	Genome sequence^b^	Calculated amplicon Tm (°C)	Measured ampicon Tm^c^ (°C)
PLRV	F	AAGAAGGCAATCCCTTCG	0.25	155	LC501445	87.5	87.6 ± 0.3
	R	ATGTCTCGCTTGAGCCTC					
PVS	F	TCGTBTGGAATTACATGCTMG	0.50	102	AB451180 (PVS^O^)	81.0	82.2 ± 0.1
	R	ATCAAATGTGTCAAAWGCGG			LC492754 (PVS^A^)	82.5	83.1 ± 0.3
PVX	F	TTCGACTTCTTCAATGGAGTC	0.11	189	AB451181	84.5	85.9 ± 0.2
	R	TCCAGTGATACGACCTCG					
PVY	F	TGAAAATGGAACCTCGCC	0.14	129	AB451181 (PVY^O^)	79.0	80.5 ± 0.4
	R	AATGTGCCATGATTTGCC			AB331515 (PVY^NA-N^)	78.0	79.4 ± 0.2
					AB702945 (PVY^NTN^)	79.0	80.5 ± 0.3
*EF1α*	F	TACTCCAAGGCTAGGTATGATG	0.22	74		74.0–75.0	
	R	TCAGGGTTGTAACCGACC					

^a^Written according to the International Union of Pure and Applied Chemistry (IUPAC)

^b^GenBank accessions (lineage for PVS or strain for PVY) of isolates used for the calculation and the measurement of Amplicon Tm values were shown, corresponding to the values in the same line. For potato *EF1α*, five sequences of mRNA (GenBank accessions DQ2288628, DQ222490, AJ536671, AB061263, and KF537426) were used

^c^Measured Tm values were shown, and the values meant “(Average Tm value) ± 2 × (Standard error).”

### Confirmation of detection sensitivity for each virus

The identical four virus isolates were used in this experiment: ChLR_2 for PLRV, M for PVS^O^, O-IC249 for PVX, and Eu-12Jp for PVY^NTN^. The detection sensitivity was confirmed using tenfold dilution series for each virus, prepared by mixing with singly-virus-infected and virus-free potato leaf homogenate. The RNA preparation, the assay, and the analysis of the result were done as described above.

## Results

### Primer design

Considering genetic diversity of PVS and PVY, primer sequences reported by Cheng et al. [12] were compared with genome sequences, and PVY primers’ sequences matched with sequences of strains but PVS primers mismatched with PVS^A^ and PVS^P^ (Additional file 1: Figure S2). The conserved regions among PVS genome sequences for coat protein gene (CP) were few because of their genetic diversity; a pairwise distance of seven sequences was 0.20 on average. For the same reason, the amplicon Tm values for PVS were varied by its lineages, and our PVS primers could not be used with their primers for the others because amplicon Tm values were overlapped with each other. Therefore, all primers for the four viruses were newly designed not to make their amplicon Tm values overlap, and the differences of the values were more than 2.0 °C (Table 2). The viral primers were targeted to CP, and PVS primers were designed based on conserved region among three lineages (Additional file 1: Figure S1). Strains of PVY are divided into six groups by their nucleotide sequences for CP, and the representative strains for the six group are C strain (PVY^C^), O strain (PVY^O^), O5 strain (PVY^O5^), Eu-N strain (PVY^Eu-N^), NA-N strain (PVY^NA-N^) and E strain (PVY^E^) [15]. The sequences for PVY primers were designed based on the conserved region among these six strains. PVX and PLRV primers were also derived from conserved regions among strains; however, their sequences for CP were highly conserved and did not divide into any groups by genome sequence. The primers were also designed to make mismatches less than two in all examined pairs of each primer and genome sequences, and degenerated nucleotides were used for only PVS primers to diminish mismatches.

### Optimization of one-step real-time mRT-PCR assay

**Fig. 1:**
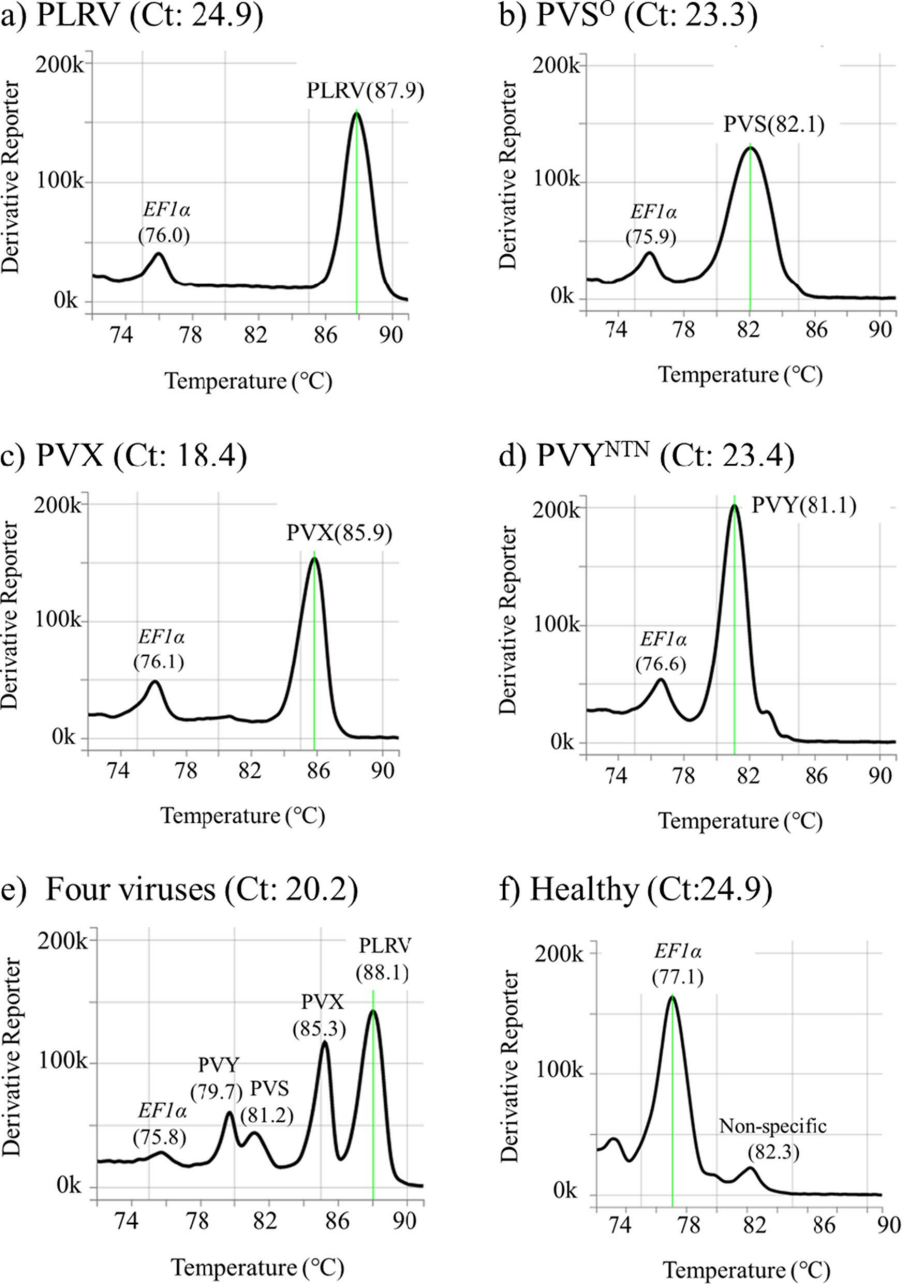
Detection of four potato viruses from total RNA extracted by simple RNA preparation. RNA solutions were prepared from virus-infected potato (cv. Irish Cobbler) leaf homogenate with **a** PLRV (isolate: ChLR_2), **b** PVS^O^ (M), **c** PVX (O-IC249), or **d** PVY^NTN^ (Eu-12Jp). Simultaneous detection of the four viruses was confirmed using **e** RNA solution prepared from the mixture of equal volume of four singly-virus-infetcted leaf homogenates. RNA solution prepared from **f** virus-free potato leaf homogenate (Healthy) was used as negative control. One-step real-time mRT-PCR was performed as described in Materials and Methods, and results analyzed by the instrument software; peaks (melting temperature) for plant *EF1*α and each virus are shown. The Ct value corresponding to each melt curve is given after the panel caption

**Fig. 2:**
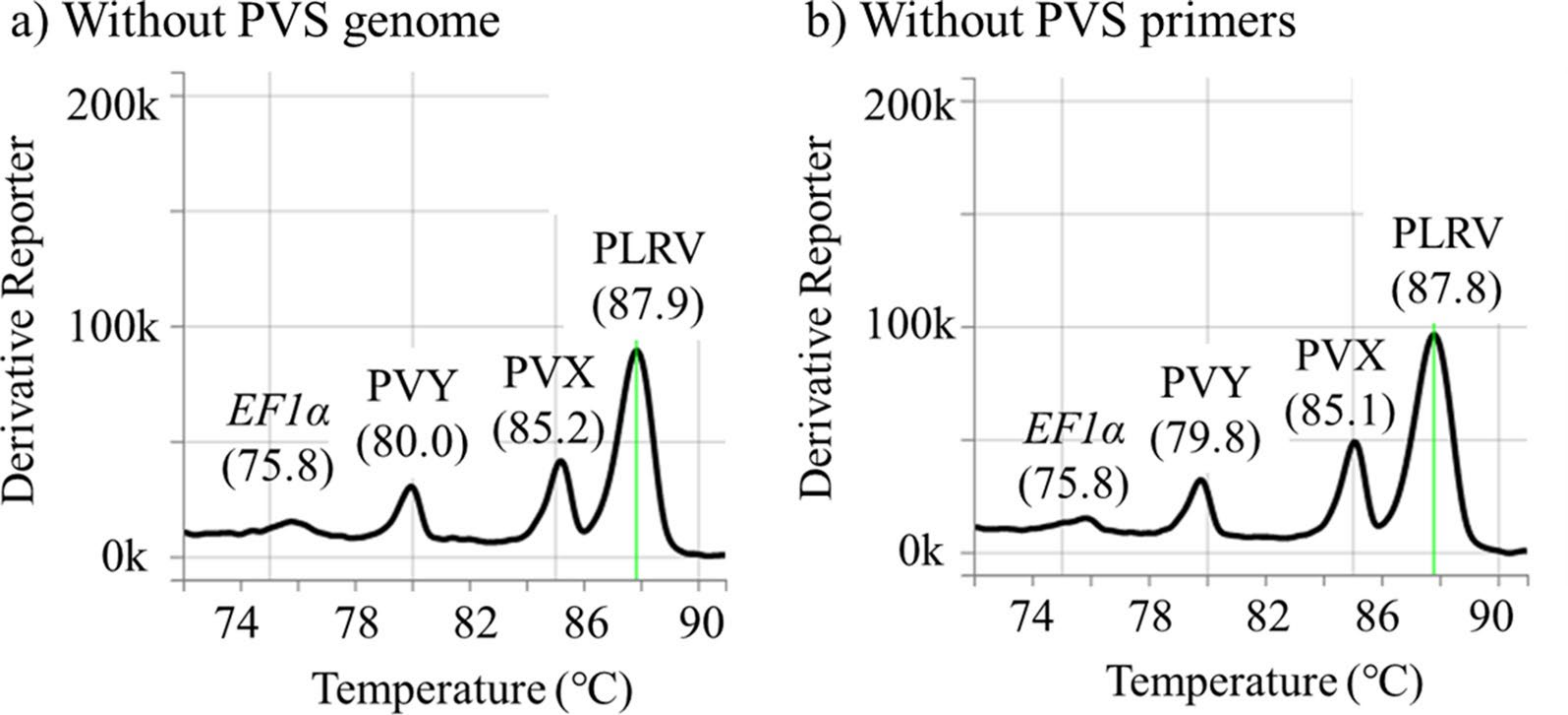
Melt curve analysis for multiple detections of four potato viruses without the PVS genome or PVS-specific primers. Using singly-virus-infected potato (cv. IC) leaf homogenates, the templates were prepared by mixing **a** the three RNA solutions of PLRV (isolate: ChLR_2), PVX (O-IC249), and PVY^NTN^ (Eu-12Jp) or from **b** the mixture of four virus-infected homogenates, PLRV, PVS^O^ (M), PVX, and PVY^NTN^. The peaks (melting temperature) for plant *EF1*α and each virus are shown

Primer concentrations in the reaction mixture were optimized to simultaneously detect the four viruses and diminish non-specific peaks in the melt curve (Table 2). With optimized primer concentrations, our assay solely detected each virus from singly-virus-infected potato leaf homogenates, and the threshold cycle (Ct) values were less than 25.0, and Tm values for targets were distinct from each other; 87.9 °C for PLRV, 82.1 °C for PVS, 85.9 °C for PVX, 81.1 °C for PVY and from 75.9 to 76.6 °C for *EF1α* (Fig. 1a–d). Our assay also detected the four viruses simultaneously, and the peaks for viruses were distinct from each other (Fig. 1e). Although the peak for PVS was still lower than that of the other viruses in simultaneous detection, applying more than 0.50 µM of PVS primers did not improve the peak size. A non-specific peak around 82.0 °C was sometimes observed in the melt curve of virus-free potato leaf samples (Fig. 1f), and this phenomenon was also not improved by any change in the primer concentrations. The non-specific peak might lead to a false positive for PVS, especially during simultaneous detection of four viruses, so we verified this possibility (Fig. 1e). Melt curve analysis of multiple-virus detection without the PVS genome or the PVS-specific primers showed no peak around 82.0 °C, indicating that the peak of 81.2 °C in simultaneous detection was derived from the PVS genome and the non-specific peak around 82.0 °C would be caused by a primer dimer (Fig. 2).

Then, a viral diagnosis of non-Japanese isolates was presumed by the calculation. The accuracy of calculation was validated by comparing calculated Tm values with measured Tm values for Japanese isolates (Table 2). These values had a strong linear correlation (*R*^2^ = 0.9875), and the calculated Tm value seemed to be sufficiently reliable to presume Tm values for non-Japanese isolates using their genome sequences. Based on the calculation, Tm values for representatives were not overlapped with other viruses, and the values for PVY were from 77.5 to 79.5 °C, PVS from 81.0 to 82.5 °C, PVX from 84.5 to 85.5 °C, and PLRV from 87.0 to 87.5 °C in ascending order (Table 3). The validated calculation indicated that our assay could diagnose representative non-Japanese isolates of four viruses, including three lineages of PVS and six strains of PVY.

**Table 3:** Calculated amplicon Tm values of representative non-Japanese isolates

Virus	Isolate	Accessions^a^	Strain/lineage^b^	Calculated Tm (°C)
PLRV	PLRV165	MG356502	–	87.0
	PLRV-IM	KC456052	–	87.0
	14.2	AF453394	–	87.5
	D00530	D00530	–	87.5
	GAF318-4.2	KU586454	–	87.5
	Polish isolate	X74789	–	87.5
PVS	Leona	AJ863509	PVS^O^	81.0
	Dic2	KR152654	PVS^P^	81.0
	BB-AND	JQ647830	PVS^A^	81.5
	RL5	JX683388	PVS^A^	81.5
	Id4106-US	FJ813513	PVS^O^	82.0
	RVC Andean	JX419379	PVS^P^	82.0
	Yunnan YN	KC430335	PVS^O^	82.5
PVX	X3	D00344		84.5
	Taiwan	AF272736	–	84.5
	PVX_Tn148	MF682528	–	84.5
	FX21	EF423572	–	85.0
	Iran	FJ461343	–	85.0
	JAL-2	KR605396	–	85.5
PVY	NC57	DQ309028	PVY^C^	77.5
	Adgen	AJ890348	PVY^C^	78.0
	P141	MT264735	PVY^NA-N^	78.0
	RRA-1	AY884984	PVY^NA-N^	78.0
	PVY-MON	JF928458	PVY^E^	78.5
	PVY-AGA	JF928459	PVY^E^	79.0
	CO11	KY847937	PVY^O5^	79.0
	MT100010	KY847987	PVY^O5^	79.0
	MSU_45_384a	KY847984	PVY^Eu-N^	79.0
	MT100017	KY847988	PVY^Eu-N^	79.0
	ME_236_120	KY847962	PVY^O^	79.0
	CA14	KY847936	PVY^O^	79.5

^a^GenBank accession

^b^The classification of the lineage for PVS was according to Vallejo et al. [14], the strains for PVY according to Green et al. [15]

### Confirmation of detection sensitivity to each virus

**Fig. 3:**
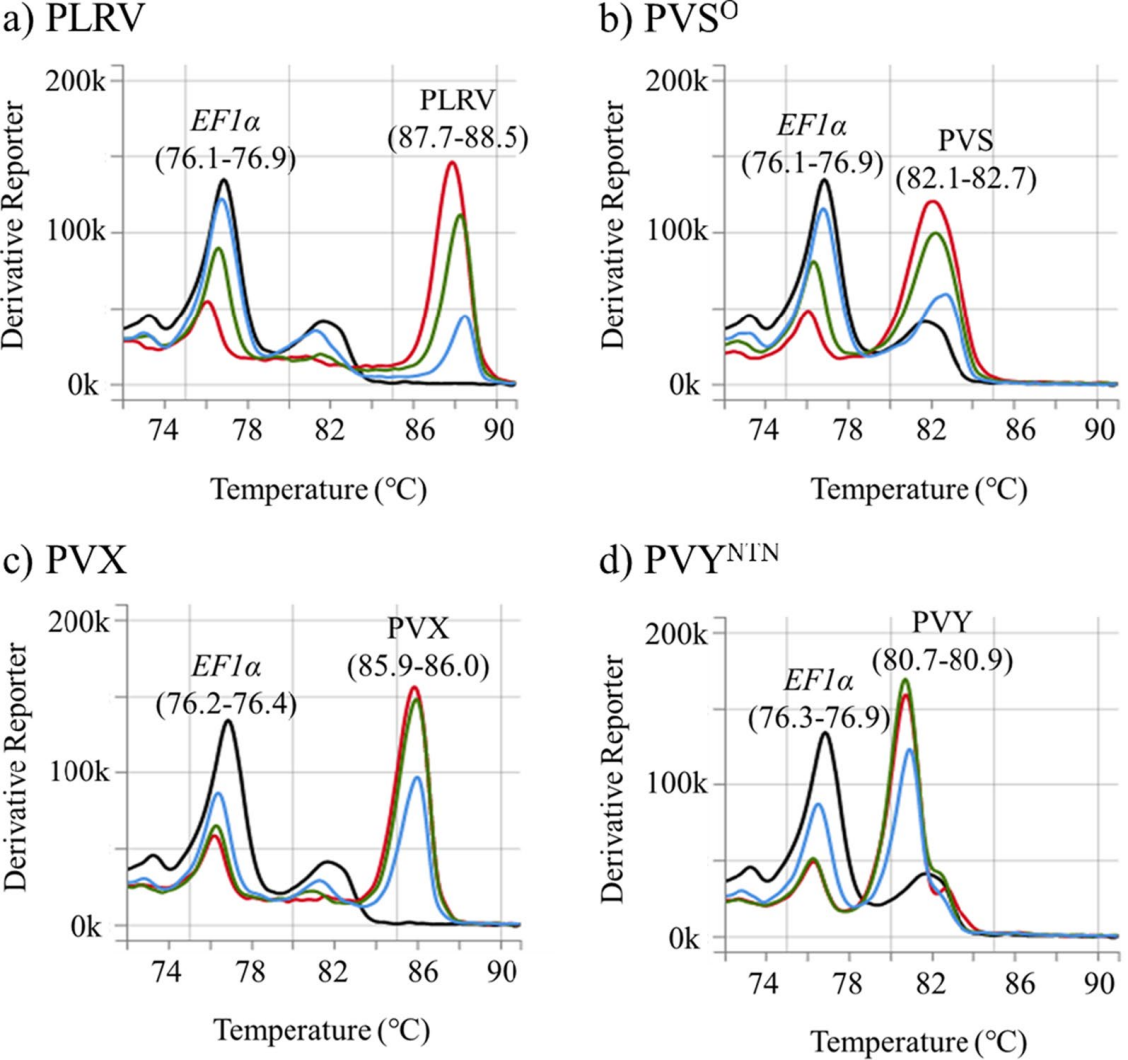
Confirmation of detection sensitivity for each potato virus. Ten-fold dilution of leaf homogenates was prepared by mixing virus-free and virus-infected potato leaf homogenates with **a** PLRV (isolate: ChLR_2), **b** PVS^O^ (M), **c** PVX (O-IC249), or **d** PVY^NTN^ (Eu-12Jp). The peaks (melting temperature) for plant *EF1*α and each virus are shown. Melt curve colors: black, virus-free potato leaf; red, a tenfold dilution of each virus; green, 100-fold of each; blue, 1000-fold

The detection sensitivity to each virus was confirmed using dilution series of singly-virus-infected potato leaf homogenates. The specific peaks for each virus were observed from tenfold dilution to 1000-fold dilution (Fig. 3). The specific peaks for PLRV, PVX, and PVY in all dilutions were distinct from a non-specific peak around 82.0 °C, and our assay could diagnose these three viruses until 1000-fold dilution. The PVS-specific peak in a 1000-fold dilution was observed; however, difficult to distinguish with a non-specific peak around 82.0 °C in virus-free homogenates and lower than the *EF1α*-specific peak in the same sample. Therefore, the detection sensitivity to PVS was under 1000-fold dilution.

## Discussion

For the seed potato certification program needed to test the numerous pathogens that can infect potatoes, high-throughput detection methods are necessary to test many tubers. A real-time PCR assay is a candidate for the assay to save more labor for viral detection than a conventional PCR assay or ELISA. An assay using fluorescent probes has a great advantage on multiple detections against an intercalator method because the wavelength of fluorescent identifies the target. However, probes are additionally designed based on conserved regions. PVS genome sequences have high diversity, and it is difficult to design a probe in the range of 80–150 bp, which is a suitable amplicon size for real-time PCR. Therefore, a method using fluorescent DNA dye, a real-time mRT-PCR assay with melt curve analysis, was selected as a development objective in this study.

There was no real-time RT-PCR assay with melt curve analysis which would detect all Japanese isolates of the four viruses. All primers for the four potato viruses were newly designed in the conserved region among representatives not to overlap amplicon Tm values with the other species to meet our requirement. Amplicon Tm values for PVS and PVY were within a broad range and varied by lineages of PVS and strains of PVY, from 82.2 (PVS^O^) to 83.1 °C (PVS^A^) and from 79.4 (PVY^NA-N^) to 80.5 °C (PVY^O^ or PVY^NTN^), respectively. This result was consistent with the previous study on real-time mRT-PCR assay with melt curve analysis for four grapevine viruses, and the values for each virus species were also varied about 1.0 °C according to isolates in their report [17]. We could not obtain all lineages of PVS and strains of PVY, so the variability of Tm values was confirmed by calculation. Calculated Amplicon Tm values for representatives were not overlapped with other viral species, and the difference was more than 1.5 °C; the minimum was between isolate CA of PVY^O^ and isolate Leona of PVS^O^. The actual Tm values for these isolates in our assay were unknown, and the Tm values for these isolates might be more closed to each other than our calculation. The shape of the peak for PVY in the melt curve was sharper than that for PVS; this feature would be useful for diagnosing these two viruses. Our primers would be able to identify representatives by Tm values and the shape of the peak.

Our assay could detect the four viruses simultaneously from the mixture of four homogenates of singly-virus-infected potato leaves using newly designed primers. A few real-time mRT-PCR assays with melt curve analysis for plant viruses were reported [12, 17–19], and all of them separately performed reverse transcription reaction and real-time multiplex PCR. According to our knowledge, this is the first report of a one-step real-time mRT-PCR assay with melt curve analysis for plant viruses performed. The advantages of one-step RT-PCR, which the performed reverse transcription reaction and PCR continuously in a single tube, are reducing pipetting steps, and this feature results in saving labor and preventing cross-contamination among samples. On the other hand, as a drawback, it is known that the forming of primer dimers increases. The non-specific peak around 82.0 °C was remained after optimization of the condition and could not be reduced by any modification. Fortunately, the height of the non-specific peak was lower than that of *EF1α*, so we would suggest the below criterion removed the non-specific peak. The Ct value for RNase-free water as the negative control was over 30.0, so the criterion for successful amplification was also set. Namely, these two criteria were set for our assay.

1. Successful amplification is “Ct value ≤ 30.0.”
2. Virus-specific peak higher than the internal-control peak is evaluated as positive

The two criteria also affect the evaluation of detection sensitivity. The detection sensitivity of real-time RT-PCR assays has been confirmed using purified genome RNA [11] and plasmid DNA [12, 17, 19]. Our purpose is the development of high-throughput detection protocol for seed potato quarantine. As RNA purification needs more labor than the preparation of a sample for ELISA, a simplified method was desirable. This was why crude RNA solution, which also contained DNA, prepared from dilution series of virus-infected potato leaf homogenates were used to confirm detection sensitivity to each virus. Our *EF1α*-specific primers do not cross exon and detect both mRNA and genomic DNA for *EF1α*. The peak for *EF1α* in melt curve analysis will somehow overestimate if crude RNA solution is applied as a template. The result showed that the specific peaks for all four viruses were observed in 1000-fold dilution. However, the 1000 dilution of PVS and PLRV were evaluated as negative by the second criterion set above. Our assay with criteria could detect any of four viruses from 100-fold dilution and seemed to have enough sensitivity to detect each virus in Japanese seed potato quarantine.

In Japan, plants are grown out from tubers, and the leaves of the plants are used for viral detection because the virion of PVY, which is the most common virus in the world, is gradually degrading during the dormancy period of tubers until breaking the dormancy [20]. Our organization tests nearly one hundred thousand tubers annually to confirm the viral contamination level of the potato seed lot. The level is important for grading seed potato lots in seed potato quarantine; the contamination level of a higher-grade potato seed lot is less than 1.0%, similar to other advanced countries [7–9]. Therefore, a screening method, in which samples are first bulked and screened, is effective for seed potato quarantine. According to our estimation, if the contamination level is less than 1.0%, the total number of test samples could be minimized by testing 10 to 100 individual samples in a bulked sample. Therefore, our assay has enough sensitivity to each virus for this screening. The use of this newly developed high-throughput assay would represent great savings in time and costs for labor.

## Conclusion

Our labor-saving high-throughput detection assay for internationally important potato viruses PLRV, PVS, PVX, and PVY can be used with the simple RNA preparation method we reported previously. This protocol is useful for detecting viruses in many samples for seed potato quarantine and field surveys.

## Supplementary Information

**Additional file 1. Supplementary Figure 1**. The primer binding position in viral genome. The position referred to the representative sequences written in Virus Taxonomy ninth edition: GenBank accession X14600 for PLRV, AJ863509 for PVS, D00344 for PVX, and AJ890348for PVY. Primer binding sequences and their direction were shown under each alignment, and red letters meant the position of mismatches between primer and genome sequences, and degenerated nucleotides were replaced with represented nucleotides. Abbreviations for lineages of PVS; O, PVSO; A, PVSA; P, PVSP, and those for strains of PVY; C, PVYC; NA-N, PVYNA-N; E, PVYE; O5, PVYO5; Eu- N, PVYEu-N; O, PVYO.** Supplementary Figure 2**. The confirmation of mismatches between viral genome and reported primers. The position referred to the representative sequences written in Virus Taxonomy ninth edition: GenBank accession AJ863509 for PVS and AJ890348 for PVY. Pimers’ binding sequences and their direction were shown under each alignment, and red letters meant the position of mismatches between primer and genome sequences and degenerated nucleotide was replaced with represented nucleotide. Abbreviations for lineages of PVS; O, PVSO; A, PVSA; P, PVSP, and those for strains of PVY; C, PVYC; NA-N, PVYNA-N; E, PVYE; O5, PVYO5; Eu-N, PVYEu- N; O, PVYO.

## Data Availability

Not applicable.

## Abbreviations

Contamination level: A certain proportion of tubers are randomly sampled from a seed potato lot, then assayed for potato viruses. Contamination level is the proportion of virus-positive samples among the total samples
Ct: Threshold cycle
DMSO: Dimethyl sulfoxide
dsDNA: Double stranded DNA
*EF1α*: Elongation factor 1 alpha gene
ELISA: Enzyme-linked immunosorbent assay
mRT-PCR: Multiplex reverse transcription-polymerase chain reaction
PLRV: Potato leafroll virus
PVA: Potato virus A
PVS: Potato virus S
PVX: Potato virus X
PVY: Potato virus Y
ssDNA: Single stranded DNA
Tm: Melting temperature
