# Supplementary material for: One-step real-time multiplex reverse transcription-polymerase chain reaction assay with melt curve analysis for detection of potato leafroll virus, potato virus S, potato virus X, and potato virus Y
Source: Virol J. 2021 Jun 29;18:131. doi: 10.1186/s12985-021-01591-3 (PMC8243585; doi:10.1186/s12985-021-01591-3)
Supplement: Supplementary file 1 — Additional file 1. Supplementary Figure 1. The primer binding position in viral genome. The position referred to the representative sequences written in Virus Taxonomy ninth edition: GenBank accession X14600 for PLRV, AJ863509 for PVS, D00344 for PVX, and AJ890348for PVY. Primer binding sequences and their direction were shown under each alignment, and red letters meant the position of mismatches between primer and genome sequences, and degenerated nucleotides were replaced with represented nucleotides. Abbreviations for lineages of PVS; O, PVSO; A, PVSA; P, PVSP, and those for strains of PVY; C, PVYC; NA-N, PVYNA-N; E, PVYE; O5, PVYO5; Eu- N, PVYEu-N; O, PVYO. Supplementary Figure 2. The confirmation of mismatches between viral genome and reported primers. The position referred to the representative sequences written in Virus Taxonomy ninth edition: GenBank accession AJ863509 for PVS and AJ890348 for PVY. Pimers’ binding sequences and their direction were shown under each alignment, and red letters meant the position of mismatches between primer and genome sequences and degenerated nucleotide was replaced with represented nucleotide. Abbreviations for lineages of PVS; O, PVSO; A, PVSA; P, PVSP, and those for strains of PVY; C, PVYC; NA-N, PVYNA-N; E, PVYE; O5, PVYO5; Eu-N, PVYEu- N; O, PVYO. [file 12985_2021_1591_MOESM1_ESM.docx]

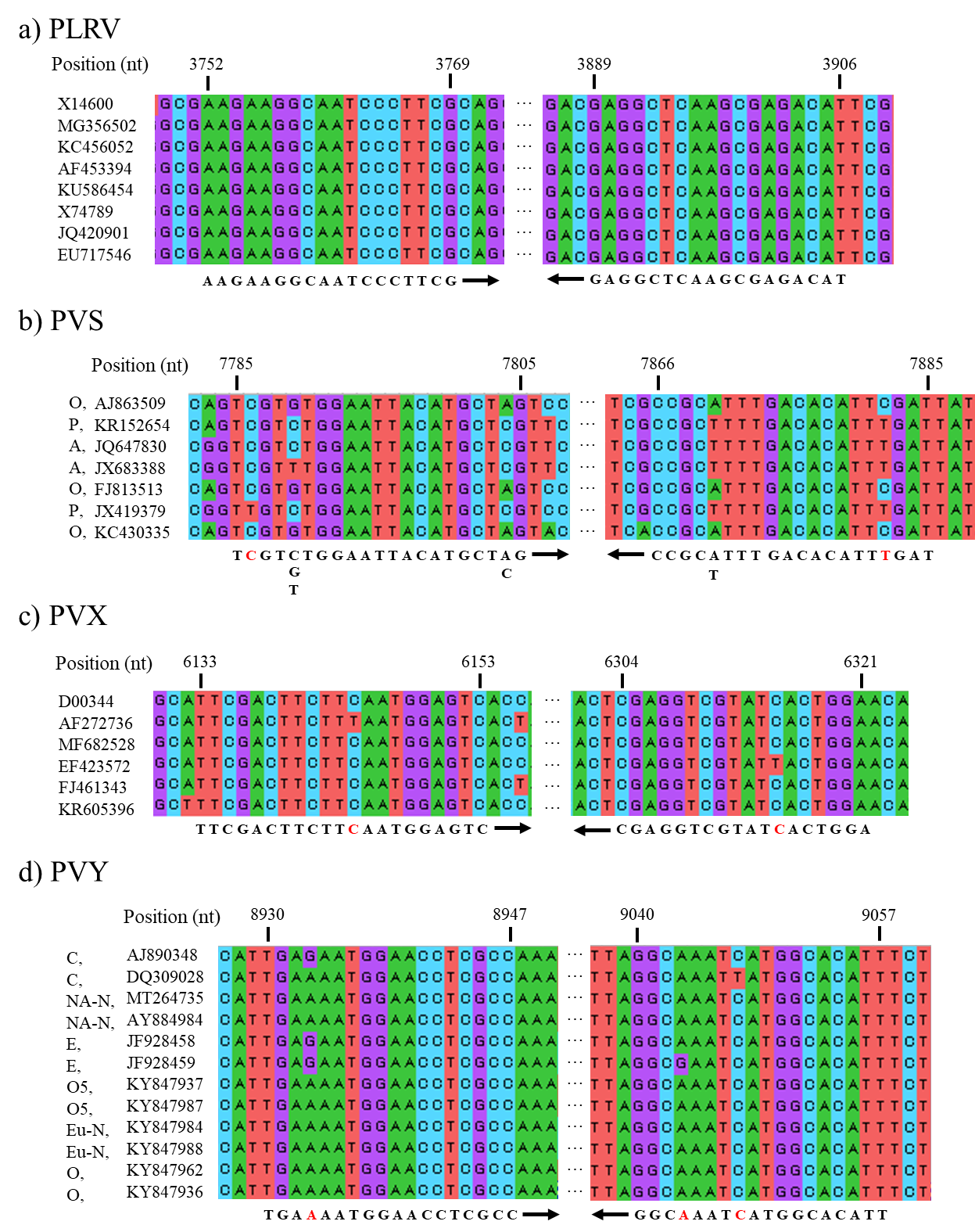


Supplementary Figure 1 The primer binding position in viral genome

The position referred to the representative sequences written in Virus Taxonomy ninth edition: GenBank accession X14600 for PLRV, AJ863509 for PVS, D00344 for PVX, and AJ890348for PVY. Primer binding sequences and their direction were shown under each alignment, and red letters meant the position of mismatches between primer and genome sequences, and degenerated nucleotides were replaced with represented nucleotides. Abbreviations for lineages of PVS; O, PVS^O^; A, PVS^A^; P, PVS^P^, and those for strains of PVY;C, PVY^C^; NA-N, PVY^NA-N^; E, PVY^E^; O5, PVY^O5^; Eu-N, PVY^Eu-N^; O, PVY^O^.


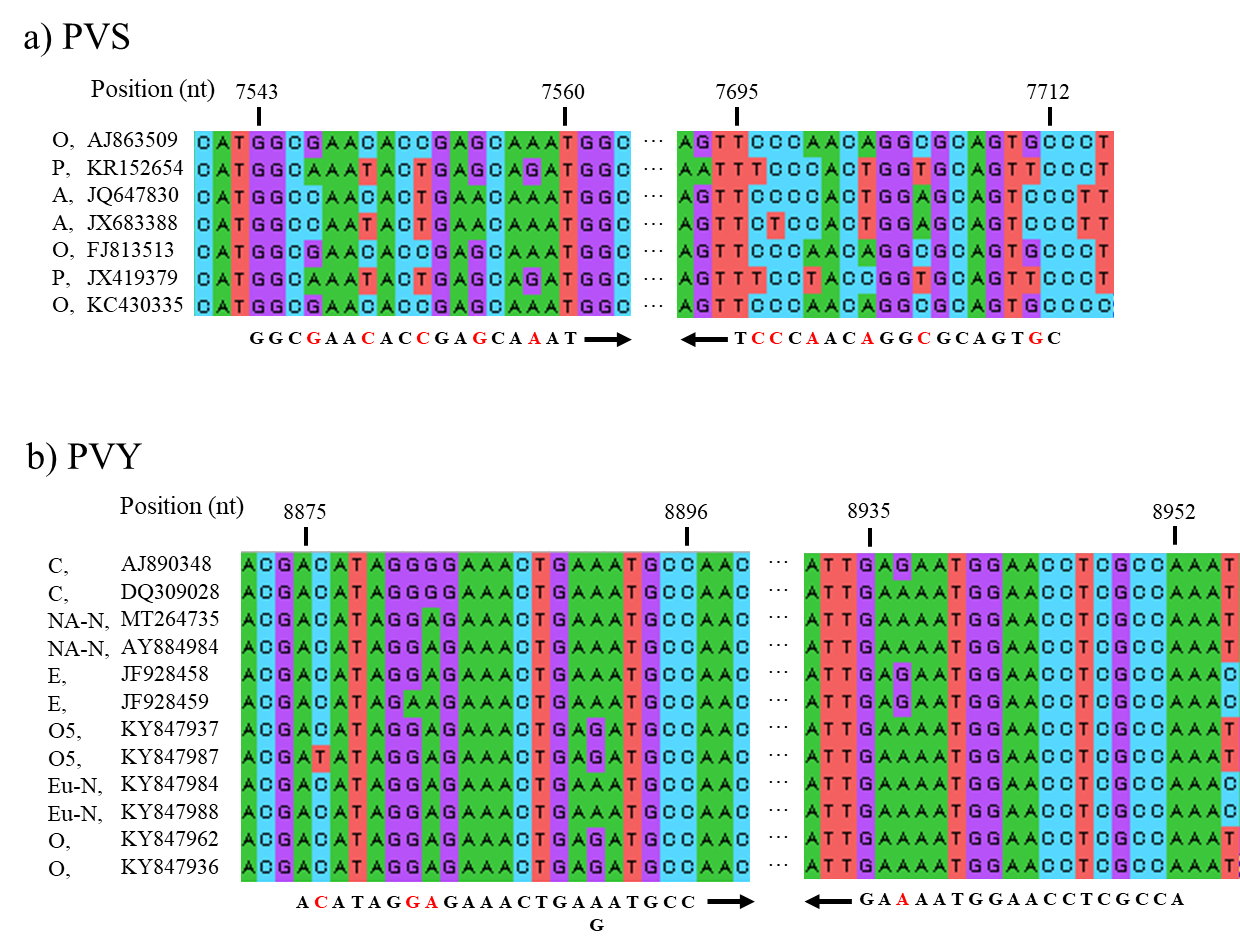


Supplementary Figure 2 The confirmation of mismatches between viral genome and reported primers

The position referred to the representative sequences written in Virus Taxonomy ninth edition: GenBank accession AJ863509 for PVS and AJ890348 for PVY. Pimers’ binding sequences and their direction were shown under each alignment, and red letters meant the position of mismatches between primer and genome sequences and degenerated nucleotide was replaced with represented nucleotide. Abbreviations for lineages of PVS; O, PVS^O^; A, PVS^A^; P, PVS^P^, and those for strains of PVY; C, PVY^C^; NA-N, PVY^NA-N^; E, PVY^E^; O5, PVY^O5^; Eu-N, PVY^Eu-N^; O, PVY^O^.
